# Sphingolipids in Gaucher disease: a systematic review

**DOI:** 10.1186/s13023-025-04015-5

**Published:** 2025-11-06

**Authors:** Ashleigh Lake, Maria Fuller

**Affiliations:** 1 Genetics and Molecular Pathology, SA Pathology at Women’s and Children’s Hospital, 72 King William Road, North Adelaide, 5006 Australia; 2 School of Biological Sciences, University of Adelaide, Adelaide, 5000 Australia; 3 Adelaide Medical School, University of Adelaide, Adelaide, 5000 Australia

**Keywords:** Gaucher disease, Sphingolipids, Ceramide, Lactosylceramide, Gangliosides, Hepatosplenomegaly, Neurological disease, Skin abnormalities, Altered sphingolipid regulation, Sphingolipid de novo synthesis

## Abstract

Gaucher disease (GD) is a rare lysosomal disorder of sphingolipid catabolism, characterised by a block in the degradation of glucosylceramide (GlcCer) to ceramide. The resulting effect is lysosomal accumulation of GlcCer and its deacylated derivative, glucosylsphingosine. Secondary alterations in the sphingolipid metabolic pathway have been reported, including elevated concentrations of ceramide, lactosylceramide (dihexosylceramide (DHC)), and gangliosides, however, there are notable inconsistencies across different cell and tissue types and their relevance to GD pathology is not well-understood. Sphingolipids are crucial for the regulation of intra- and extracellular functions and different cell types have different requirements. For example, neurons have a high demand for complex sphingolipids due to their extensive membrane remodelling networks necessary for their communication role. We therefore performed literature searches of PubMed, Scopus, and Web of Science databases to coalesce reports of sphingolipids in different animal and cell models of GD, as well as human cells and tissues from 1965 to 2024, totalling 54 studies. We found that DHC, trihexosylceramide, and simple gangliosides, G_M3_, G_M2_, G_M1_, G_D3_, and G_D2_, were elevated in most reports (79%), complex G_T_ gangliosides were largely decreased (75%), and G_D1a_, G_D1b_, and G_Q1b_ were inconsistently reported to be both increased and decreased in individual studies. Similarly, ceramide was highly discrepant between various cell and tissue types: spleen ceramide was elevated in two of three reports, brain ceramide was largely unchanged (82%), and ceramide concentrations in the skin were not consistent by any variable, including assay technique or GD sub-variant. Some of these discrepancies may be explained by biological variability and differences in methods used to measure sphingolipids, mass spectrometry being the most common, but it is clear that there are sphingolipid alterations in GD, which likely contribute to tissue-specific pathology. Evidence that sphingolipid metabolic regulation is variable across cells and tissues highlight the importance of characterising individual sphingolipid profiles on a model-to-model basis as a driving force behind cell pathology.

**Supplementary Information:**

The online version contains supplementary material available at 10.1186/s13023-025-04015-5.

## Background/review objectives

**Fig. 1:**
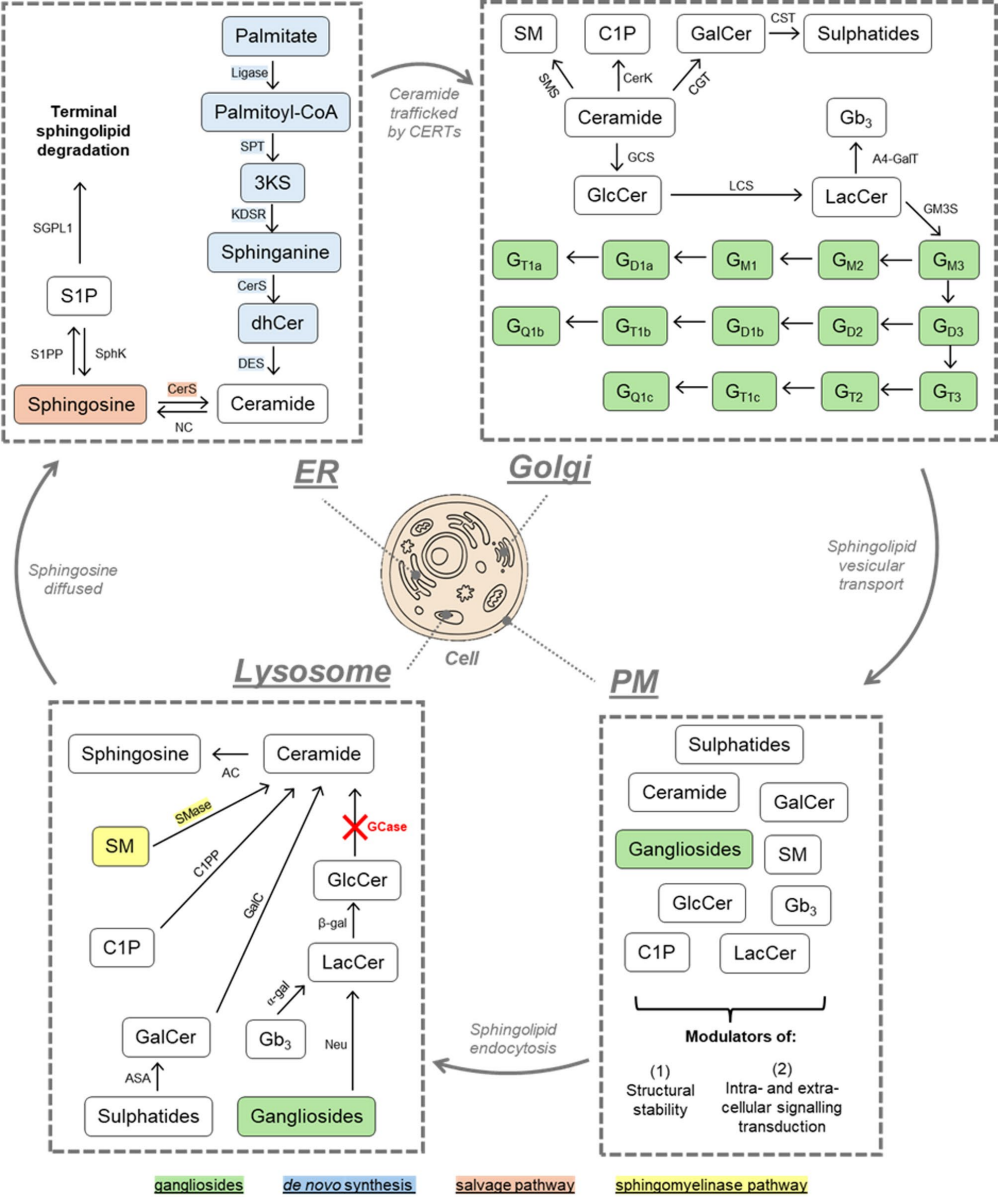
Sphingolipid metabolism in the cell. Sphingolipid de novo synthesis occurs at the endoplasmic reticulum, beginning with the activation of palmitate and concluding at the desaturation of dihydroceramide (dhCer) to ceramide. Ceramide is transported to the Golgi by vesicular transport or via the ceramide transport proteins where it may be converted to sphingomyelin (SM), ceramide-1-phosphate, glucosyl- or galactosylceramide. Glucosylceramide (GlcCer) is further metabolised to higher order sphingolipids, including the complex gangliosides. Sphingolipids are then vesicularly trafficked from the Golgi to the plasma membrane. Degradation of sphingolipids follows the same stepwise pathway of metabolism, but in reverse, and is mediated by the endolysosomal network. SM may be degraded at the plasma membrane/Golgi apparatus or in the lysosome by neutral and acid sphingomyelinase, respectively. Ceramide is then reduced to its sphingosine base at the plasma membrane or lysosome via neutral or acid ceramidase, respectively, wherein sphingosine may be reacylated back to ceramide in what is known as the salvage pathway or terminally degraded through sphingosine-1-phosphate. Red cross indicates lysosomal GlcCer catabolic deficiency in Gaucher disease by dysfunctional acid beta glucocerebrosidase. *ER,* endoplasmic reticulum; *PM* plasma membrane; *SPT,* serine palmitoyltransferase; *KDSR,* 3-ketodihydrosphingosine reductase; *CerS,* ceramide synthase; *DES,* dihydroceramide desaturase; *S1PP,* sphingosine-1-phosphate phosphatase; SPGL1, sphingosine-1-phosphate lyase 1;*SphK,* sphingosine kinase; *NC,* neutral ceramidase; *CERT,* ceramide transport protein; *SMS,* sphingomyelin synthase; *SMase,* sphingomyelinase; *GalC,* galactosylceramidase; *GCase,* β-glucocerebrosidase; *CGT,* ceramide glucotransferase; *ASA,* arylsulphatase A; *CST,* cerebroside sulphotransferase; *C1PP,* ceramide-1-phosphate phosphatase; *CerK,* ceramide kinase; *GCS,* glucosylceramide synthase; *LCS,* lactosylceramide synthase; *β-gal,* β-galactosidase; *α-gal,* α-galactosidase; *A4GalT,* α-1,4-galactosyltransferase; *GM3S,* G_M3_ synthase; *AC,* acid ceramidase; *Neu,* neuraminidase; *Gb*_*3,*_ globotriaosylceramide; *S1P,* sphingosine-1-phosphate; *C1P,* ceramide-1-phosphate; *3KS,* 3-ketodihydrosphingosine; *GalCer,* galactosylceramide; *LacCer,* lactosylceramide. Image created using Canva.com

Given that the product of glucosylceramide (GlcCer) catabolism is ceramide, it is reasonable to assume that cells will produce less ceramide in Gaucher disease (GD), as GlcCer catabolism is impaired by defective acid beta-glucocerebrosidase (GCase) activity. However, ceramide is also generated from the degradation/recycling of other simple sphingolipids and from de novo synthesis via palmitate (Fig. 1). Ceramide and other sphingolipid alterations are commonly reported in GD, but they are inconsistent across different cell and tissue types, as well as cell and animal models of GD. Therefore, in this review, we sought to coalesce the reported sphingolipid changes in GD, addressing the issue that different cells and tissues have different sphingolipid requirements for cell function/regulation and therefore may also differ when there is a block in catabolism. We performed literature searches from 1965 to 2024 to identify patient-derived samples and in vivo or in vitro models of GD wherein whole sphingolipid profiles, ceramide, lactosylceramide, globoside, or ganglioside data were reported, either in the main text or supplementary material. We also included investigations that commented on sphingolipids within models, but did not include raw data (i.e. data not shown). Following GD clinical manifestations of hepatosplenomegaly, neurological involvement, and skin abnormalities, we summarise the sphingolipid changes in GD respective to the spleen, liver, skin, and brain. Lastly, we address the potential cause of discrepant sphingolipids in GD, namely, the upregulation of one or more compensatory metabolic pathways in response to sphingolipid dyshomeostasis. The likely impact of a block in sphingolipid catabolism on downstream signalling regulation, including cell death, hyperinflammation, and developmental pathways is also discussed.

## Sphingolipid synthesis and degradation

Sphingolipids are amphipathic bioactive molecules, critical for membrane structural integrity and proper regulation of intracellular signalling [1]. Their synthesis and degradation occurs in a step-wise series of interconnected anabolic and catabolic reactions, governed by more than 40 distinct enzymes and cofactors [2]. Ceramide is a key sphingolipid, serving as the backbone for all other sphingolipid derivatives, and comprises a sphingoid base that is amide-linked to an acyl chain varying in both carbon length and saturation [3]. Ceramide is de novo generated in a series of terminal anabolic reactions at the endoplasmic reticulum (ER) (Fig. 1). At the conclusion of sphingolipid de novo synthesis, ceramide is trafficked by ceramide transfer protein (CERT) or by vesicles to the Golgi apparatus for higher order sphingolipid modification (Fig. 1). Hydroxy-linkage of varying head groups, including glucose, galactose, phosphocholine, and phosphate, at the polar end of the ceramide base produces different sphingolipid derivatives: GlcCer, galactosylceramide (GalCer), sphingomyelin (SM), and ceramide-1-phosphate (C1P), respectively. GalCer can be sulphated at the luminal face of the Golgi membrane to form sulphatide, whereas GlcCer is galactosylated at the *cis*- golgi membrane to form lactosylceramide (LacCer (dihexosylceramide, DHC)), which then serves as the direct precursor to all complex sphingolipids, including the globosides and the gangliosides. Gangliosides are complex sphingolipids which are highly expressed in the brain, and are differentiated by the number of negatively-charged sialic acid head groups at the LacCer base; the a-, b-, and c-series possessing 1, 2, and 3 sialic residues, respectively [4]. Following their synthesis, sphingolipids undergo vesicular trafficking from the Golgi to the plasma membrane (PM) where they function as modulators of structural stability, and intra- and extra-cellular signalling cascades [2].

Sphingolipid catabolism occurs in the endo-lysosomal system; sphingolipids in the plasma membrane are endocytosed and fused into the lysosome where they are sequentially degraded by acid hydrolases [1]. The catabolism of complex sphingolipids follows the same interconnected pathway of sphingolipid metabolism but in reverse, whereby each sphingolipid is stepwise reduced to its ceramide base (Fig. 1). In the lysosome, ceramide can be further reduced to its structural sphingosine base and a free acyl chain by acid ceramidase. The hydrophilic nature of sphingosine allows for its translocation from the lysosome to the cytoplasm/ER where it can be either re-acylated to form ceramide in the salvage pathway or phosphorylated to form sphingosine-1-phosphate (S1P) which may then be irreversibly degraded by S1P lyase, serving as a key exit point for complete sphingolipid degradation. ER ceramide synthesised de novo, can also be degraded by neutral ceramidase to sphingosine which may then be further metabolised to S1P or converted back to ceramide, re-entering the sphingolipid metabolic pathway. A neutral sphingomyelinase (SMase) also resides at the PM where it catalyses the degradation of SM to ceramide and a phosphocholine residue in what is known as the SMase pathway [1].

## Gaucher disease

**Fig. 2:**
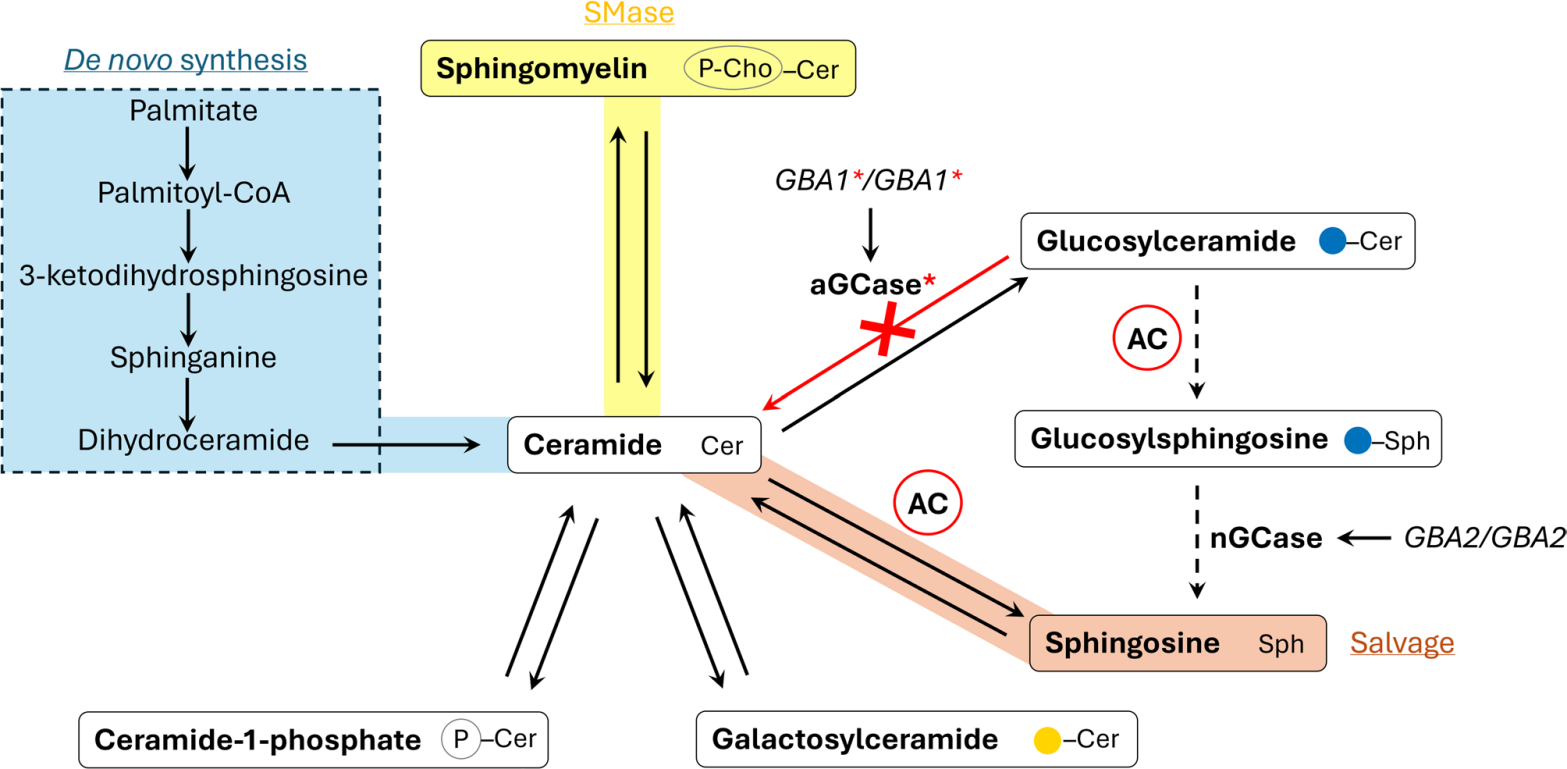
Gaucher disease cells alter sphingolipid metabolism. Biallelic pathogenic variant inheritance in *GBA1* indicates primary genetic cause of Gaucher disease (GD), resulting in a dysfunctional (lysosomal) acid glucocerebrosidase (GCase), indicated with red asterisks. Red cross/arrow depicts the resulting block in catabolism of glucosylceramide to ceramide and glucose, leading to glucosylceramide accumulation. Acid ceramidase (AC), which normally acts on ceramide to produce sphingosine, cleaves the fatty acyl chain from accumulated glucosylceramide to produce glucosylsphingosine; glucosylsphingosine is diffused into the cytosol where a neutral GCase (*nGCase*, encoded by *GBA2*) cleaves the glucose residue to form sphingosine, indicated by dashed arrows. Ceramide metabolism is also compensated by increasing de novo synthesis (blue shading). Other potential pathways of altered ceramide metabolism, sphingomyelinase (SMase) and salvage pathways, are highlighted in yellow and orange, respectively. Ceramide-1-phosphate and galactosylceramide catabolism may also serve to regulate ceramide metabolism in GD. Cer, ceramide; Sph, sphingosine; (Blue circle) glucose; (Yellow circle) galactose; (P) phosphate; (P-Cho), phosphocholine

GD is the third most common inborn error of sphingolipid metabolism in Australia with an incidence of 1.33 per 100,000 births [5], and is characterised by biallelic pathogenic variants in *GBA1* that encodes GCase, resulting in a block in the lysosomal degradation of GlcCer to ceramide and glucose [6] (Fig. 2). Although often classified into three subtypes, with type 1 referred to as a non-neuronopathic slowly progressing form, type 2 an acute, rapidly progressing neurological phenotype with infantile death, and type 3 a slower progressing chronic disease with neurological manifestations, in reality, GD exists along a broad spectrum of indolent disease through to perinatal lethality [6, 7]. In the era of enzyme replacement therapy (ERT), visceral disease is treatable, ameliorating hepatosplenomegaly and bone disease, improving life expectancy and quality. However, the inability of the recombinant enzyme to cross the blood-brain-barrier renders ERT ineffective for the neurologic manifestations [8, 9].

When GlcCer accumulation exceeds the lysosomal storage threshold, acid ceramidase can cleave the acyl chain to produce glucosylsphingosine (GluSph) [10, 11]. The formation of GluSph is thought to be a compensatory mechanism employed by the cell to rid excess GlcCer. Cleavage of the acyl chain from GlcCer increases water solubility and enables translocation from the lysosome to other cellular compartments: in the cytosol, a neutral beta glucocerebrosidase (encoded by *GBA2*) has been reported to cleave glucose from GluSph, which produces a pool of sphingosine that may be further synthesised to S1P or recycled back into the sphingolipid pathway [10, 12]. This is direct evidence of a self-employed mechanism by the cell to combat the block in GlcCer degradation. Although this process is seemingly beneficial, GluSph and other lyso-sphingolipids are well-established cytotoxic molecules, contributing to the cascade of cell decline and eventual death [10, 13, 14]. This raises the following questions: (1) how do individual cells sense and compensate sphingolipid metabolism in response to a violation of sphingolipid homeostasis and (2), is this altered regulation beneficial to the cell, or a self-employed pathological response.

## Methods

### Search strategy and study inclusion criteria

The systematic literature review was performed in accordance with the guidelines provided by the Preferred Items for Systematic Review and Meta-Analyses Protocols (PRISMA-P) [15]. Search terms were determined according to the Medical Subject Headings (MeSH) and were as follows: ceramide; (glycosphingolipid OR GSL); ganglioside; (lactosylceramide OR dihexosylceramide OR DHC); (globoside OR Gb_3_ OR trihexosylceramide OR THC); G_M_; G_D_; G_T_; G_M3_; G_M2_; G_M1_; G_D3_; G_D2_; G_D1_; G_D1a_; G_D1b_; G_T3_; G_T2_; G_T1a_; G_T1b_; G_T1c_; G_Q_; G_Q1b_; G_Q1c_ AND Gaucher. Records were identified from the US National Library of Medicine, inclusive of Medline and PubMed, Scopus, and Web of Science databases between 1965 and 2024 with no search period restrictions. Eligibility criteria included original research articles (excluding clinical trial reports) reporting on ceramide or complex sphingolipids in GD. Articles that did not necessarily include clinical trial data but where GD patients were receiving or had previously received either enzyme replacement or substrate reduction therapy were removed to ensure that the reported sphingolipids were not a consequence of therapeutic intervention, however, baseline (pre-therapy) data were accepted.

### Quality assessment

Risk of bias assessments were performed for all identified reports and appropriate tools selected depending on the design of the study. Twenty four observational studies were assessed with the Newcastle–Ottawa scoring tool [16] in which 19 were determined to have a high risk of bias (score 0–3)(Supp. Table 1). Eighteen in vitro analyses were assessed with the Quality Assessment Tool For In Vitro Studies (QUIN Tool) [17] and our analyses determined there to be low risk for all included reports (Supp. Table 2). In vivo studies were assessed with the SYRCLE risk of bias tool [18] and all but 1 of the 12 reports were determined to have low risk of bias (Supp. Table 3).

## Results

### Search outcomes

**Fig. 3:**
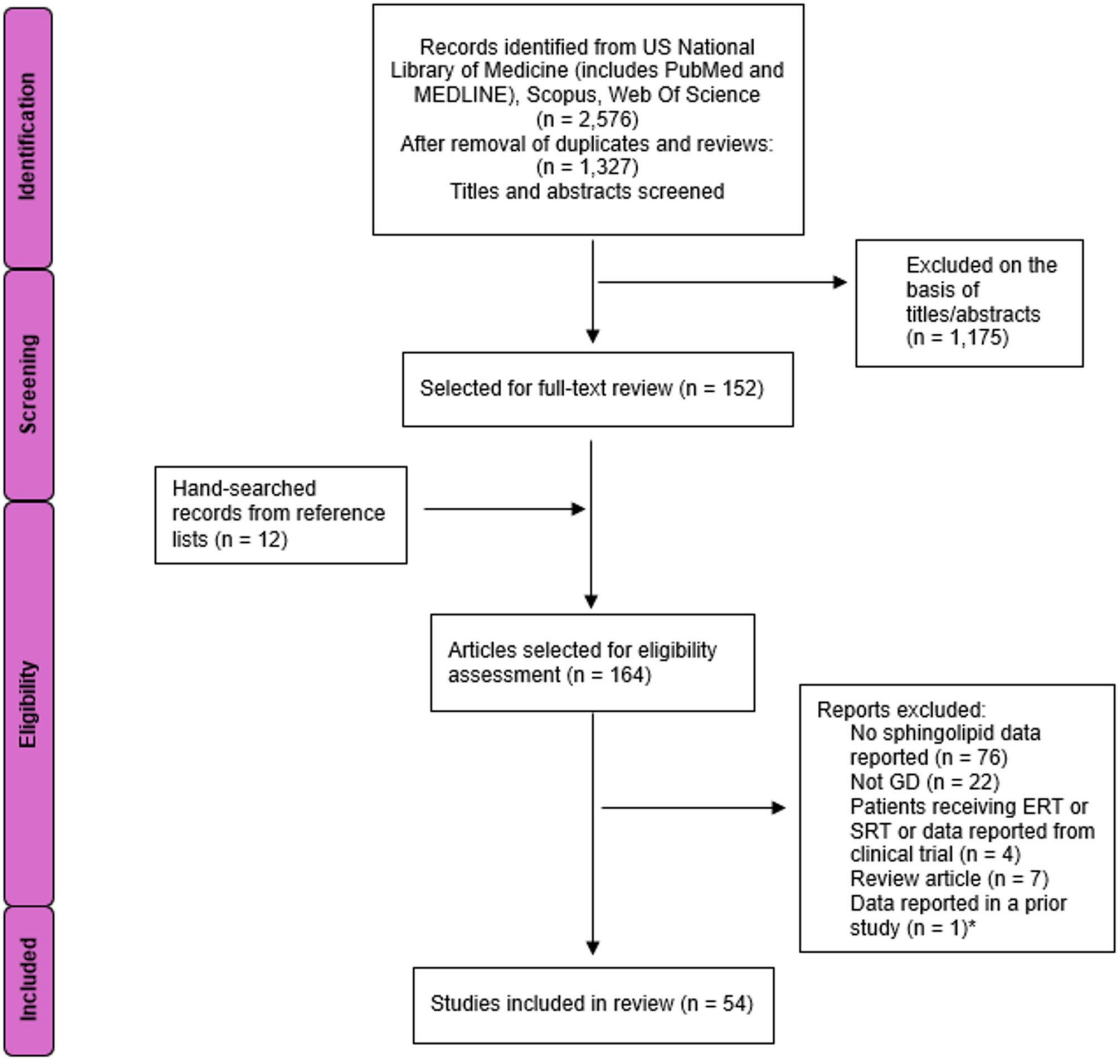
PRISMA flow diagram depicting literature identification and article selection process for publications reporting sphingolipid data in Gaucher disease (GD). ERT, enzyme replacement therapy. SRT, substrate reduction therapy. *Prior study included in review

The literature searches on sphingolipids in GD produced a total of 1,327 articles for consideration (Fig. 3). One thousand one hundred and seventy-five of these articles were removed on the basis of abstract/title screening following the study inclusion criteria described above (Methods). Additional articles were sourced from references of identified reports in the screening process and a total of 164 studies were selected for full-text review wherein 54 met the eligibility conditions. Most reports were excluded on the basis of the lack of sphingolipid data or not specifically related to GD, including reports of dysfunction/genetic ablation of the entire *PSAP* gene or the pre-cleaved prosaposin (PSAP) protein which produces widespread saposin (Sap) A-D deficiencies that impact other sphingolipid acid hydrolases [19] (Fig. 3).

### Sphingolipid alterations in GD

Of the 54 reports that measured sphingolipids in GD, totalling 67 different tissues or cells or models, the brain, skin, and spleen were the most common tissue types at 12, 12, and 9 reports, respectively. Other tissues included liver (*n* = 3) and lung (*n* = 2), and the remaining data were reported from cell/subcellular models (*n* = 17) or from liquid biopsies (e.g. blood, urine) (*n* = 12).

#### Elevated sphingolipids contribute to hepatosplenomegaly in GD

As splenomegaly is a cardinal feature of disease, the spleen was the initial focus of biochemical investigations, with studies originating over 60 years ago. Although GlcCer accumulation in the spleen is well-documented, GlcCer accumulation accounts for only 2% of the additional tissue mass [20], suggesting other factors are responsible for spleen enlargement. In five GD patients (Supp. Table 4), a 2–3 fold elevation in ceramide was reported in the spleen compared with unaffected controls [21, 22], a somewhat contraindicated finding given that ceramide is the product of GlcCer catabolism and a reduction would be empirically expected. Similar elevations of ceramide have been noted in the liver from a combined Sap C/GCase deficiency mouse model [23], but interestingly, the naturally-occurring ovine GD model exhibits normal ceramide in both the spleen and the liver [24] which may imply GD sheep are not an authentic model of human disease.

Like ceramide, more complex sphingolipids appear to be elevated in the GD spleen (Supp. Table 4). Trihexosylceramide (THC), G_M3_, G_M2_, and G_M1_ have been shown to be elevated in 29 GD patient spleens across four different reports [21, 25–27] and in different GD animal models [24] or macrophage/spleen microdomain reports [28–30]. Given the mass accumulation of GlcCer, and elevations in other downstream metabolites such as G_M3_ and THC, it might also be expected that DHC is elevated, but interestingly, DHC was unchanged in five splenic biopsy reports, encompassing 36 individual GD patients [22, 26, 27, 31, 32]. It is important to note that of these biopsied patient spleens, only one report was published in the 21st century, and even this investigation is > 20 years old. Each analysis of human spleen used thin layer chromatography (TLC) to measure DHC whereas contemporary practice is mass spectrometry analysis. These data also do not align with more recent reporting in a variety of GD models wherein spleen and liver from the GD sheep, spleen from the conduritol B epoxide (CBE)-induced mouse, and CBE- in vitro macrophages, including lysosomal membrane microdomains from the latter two, all exhibit significant elevation of DHC [24, 28–30, 33]. As such, it is difficult to assess what is real: (1) the lack of change in human splenic DHC is a result of technological disadvantage that failed to capture a complete sphingolipid profile, or (2) the five biopsy reports here, encompassing 36 different GD patients, reveal a new insight which is not reflected in contemporary disease modelling tools.

All-in-all, elevation of ceramide and other sphingolipids, secondary to GlcCer accumulation, unequivocally contribute to increased spleen and liver mass in GD. Although, it is rather unlikely that the additional sphingolipid content solely contributes to splenic enlargement, which may manifest up to 25-times the normal weight [20]. The activation of intracellular pathways, such as inflammation, and impaired molecular degradation processes such as autophagy, which have been reported in splenic-derived GD cells (macrophages), and other splenic myeloid/T cells [34, 35], probably account for the remaining additional volume.

#### GD skin manifestations are associated with elevated LacCer, and variable ceramide changes

Severe perinatal forms of GD are associated with epidermal manifestations, including ichthyosis, collodion births, and nonimmune hydrops fetalis [7], and therefore a comprehensive analysis of sphingolipid alterations in the skin was investigated. Supplementary Table 4 shows that ceramide in the skin is highly variable. Fibroblasts and epidermis from GD patient skin samples, either directly analysed or cultured in vitro, exhibit either unchanged [36–38], increased [38–41], or decreased [36, 42, 43] ceramide compared to controls. Only cases of Sap C-deficiency were linked to consistently elevated ceramide [38, 40, 41], likely via dysregulated ceramide hydrolysis by acid ceramidase wherein Sap C is also a known cofactor [19]. Atypical Sap C-GD should therefore be interpreted separately from classical GCase-deficient cases. *GBA1* pathogenic variants also do not correlate with ceramide changes in GD where one report noted elevated epidermal ceramide in N370S homozygous mice and unchanged concentrations in D409V homozygotes [38]. These results might be expected since D409V mice do not exhibit significant GlcCer accumulation, overt pathology, and have a normal life span, whereas N370S mice die within 24–48 h after birth due to skin barrier permeability [44], possibly due to ceramide imbalance. It is confounding however, that N370S mice are homozygous for an allele typically associated with type 1 disease in humans whereas the D409V variant, only having been described in human heterozygotes, is predicted to produce severe disease [44, 45]. Another two severe GD murine models, harbouring the RecNcil genotype (*GBA1* and *GBAP1* recombination) and *Gba* null alleles, both exhibit significantly reduced ceramide in the epidermis and stratum corneum, respectively [42, 43]. Reports in human skin across different GD subtypes are similarly variable; one report found that ceramide was significantly decreased in type 2 human epidermis, but unchanged in types 1 and 3 [36], another found significantly elevated ceramide in both type 1 and type 2 fibroblasts with no differences between variants [39], and a third report observed no change in type 1, 2, or 3 fibroblasts [37]. Taken together, it is unclear whether disease severity has an outcome on ceramide in GD skin, but it is clear that ceramide concentrations fluctuate, seemingly on a case-by-case basis. The inherited *GBA1* pathogenic variants and their potential contribution to ceramide alterations cannot be determined, but ceramide variability does suggest the following: (1) ceramide turnover in the skin is rapid; (2) that there is a potential mechanism/s to combat the loss of GlcCer turnover in some, but not all individuals; and/or (3) that this mechanism/s is only somewhat regulated.

Concentrations of glycosphingolipids in GD skin are more consistent (Supp. Table 4). Using TLC and liquid scintillation spectrometry, Barton et al. [46] found that globosides and DHC were significantly greater than controls in the fibroblasts of a GD case report with unspecified *GBA1* variants. Dawson et al. [47] measured G_M3_ at 1.79 and 1.47 µmoles/g in two adult GD skin fibroblasts, compared to controls at 1.09 µmoles/g and although the authors did not comment on G_M3_, concentrations did appear greater in GD patient samples. Similarly, two more recent reports of sphingolipids in GD fibroblasts (collectively, eight type 1 and seven type 2 variants) showed significant elevation of DHC, THC, and G_M1_ ganglioside compared to controls: whereas Fuller and colleagues [39] did not find any differences in DHC or THC according to disease severity, Ceni et al. [48] found that fibroblasts from a N370S homozygote (type 1) had unchanged G_M1_, but in severe cases (L444P homozygote and R131C homozygote) had up to 5-fold and 110% increased G_M1_, respectively. Only one, 40-year-old study reported an in-depth TLC analysis of DHC, G_M3_, G_M2_, G_M1_, G_D3_, G_D1a_, and globosides and found all resided within the normal range [49]. The lack of change in complex sphingolipids, particularly the gangliosides, is not completely unexpected as they reside primarily within the brain [4]. In conclusion, GD skin appears to present with a distinct pattern of elevated simple sphingolipids, consistent with their expression levels throughout the body.

#### Neuronopathic GD is associated with normal ceramide, elevated simple gangliosides, and reduced complex gangliosides

The neuronopathic phenotypes of GD and the high sphingolipid content of the brain has precipitated 11 reports quantifying ceramide in GD neural cells and tissues. Three reports of whole brain analyses from GD mice [23, 50, 51] and one report of brain analysis from GD sheep [24] show unchanged ceramide compared to controls. Additional in vitro neuronal models of GD, either GBA1 knockdown, CBE-induced, and one report of microdomains from GD sheep brain, also report unchanged ceramide (71%) [33, 52–55]. Two reports show decreased ceramide in CBE-treated murine cerebellar granule neurons [55], and in *GBA1* knock-out SK-N-SH neuroblastoma cells [56], but overall, there is no clear trend of regional or neuronal phenotype-specific ceramide change in the GD brain. Preserved ceramide concentration in the brain might reflect a lesser need for neural GlcCer turnover or may indicate a well-controlled mechanism to counter impaired GlcCer degradation. On the other hand, DHC is consistently elevated in GD type 2 brain (*n* = 5–6) [27, 57] and in neuronopathic animal models of GD, including sheep and mice [24, 50, 58, 59], which could indicate further anabolism of GlcCer and the cells’ intrinsic requirement for more complex sphingolipids for neural function.

Gangliosides have been of significant interest to researchers since the early 1970s, given their predominance in the brain and importance in neurological development and function. Six individual autopsy analyses, encompassing 20 GD patients (Supp. Table 4) reported a general trend of elevated simple gangliosides, G_M3_, G_M2_, G_M1_, and significant reductions of the more complex gangliosides, G_T_ and G_Q_, that was somewhat distinguishable by GD subtype. Only one report measured ganglioside concentrations from whole brains of two type 1 GD cases and found concentrations unchanged compared to controls [25]. Similarly, type 3 GD (*n* = 6–8) and an atypical case of type 1/3 all exhibited elevated brain (cerebral and cerebellar cortices) globosides with normal ganglioside composition [27]. Acute neuronopathic cases (type 2) however, exhibit consistently elevated levels of globosides and G_*M2*_ in the cerebral (*n* = 4) and cerebellar cortices (*n* = 5) [27], elevated G_*M3*_ consistent across whole or regional analysis (*n* = 6–7) [27, 60, 61], and increased G_*M1*_ and G_*D1b*_ from patient whole brain [60, 62]. One GD type 2 study and a case of atypical Sap C-GD also noted moderately increased G_*M1*_ and reduced G_*T*_ gangliosides across both grey and white brain matter [62]. Although further reports are required for complete comprehensive analyses, ganglioside changes do not appear region-specific, but rather, correlate with severity of disease. Neuronopathic GD animal models, such as the GD sheep or genetically/chemically induced GD mice, also displayed similar ganglioside changes, consistent with the rapid progressing type 2 GD phenotype. Notably, G_M3_, and G_M1_ were elevated [24, 50, 58, 59], G_M2_, G_D3_, G_D2_, and G_D1a_ were increased in another two reports [24, 59], and reduced concentrations of G_D1b_ and G_T_ gangliosides were seen in another [58]. Results were also consistent across whole or regional (cortex/sub-cortex [59] and hippocampus/midbrain [24]) brain analyses. Supplementary Table 4 also reveals a further four in vitro models of CBE-induced neuronopathic GD with reported sphingolipid changes: radio-isotope labelling of hippocampal neurons did not reveal any significant differences of labelled-sphingolipids [53], whereas G_M2_ and G_M3_ were elevated, with a concomitant reduction of G_Q1b_, in GD cerebellar granule neurons [55]. Conversely, GD dopaminergic neurons from this same report [55] noted significantly elevated G_M3_, G_M1_, G_D1a_, G_D1b_, and interestingly, an increase in G_Q1b_. These results suggest that the observed sphingolipid profile may vary depending on the biological phenotype of the neuron (e.g. cholinergic/dopaminergic/glutamatergic subtypes), which likely have distinct sphingolipid requirements for proper function. Accordingly, regional analyses of gangliosides, rather than whole-brain measurements, may offer a more nuanced insight into GD neuropathology. Only one study measured sphingolipids in a cell outside of the neuron: Gregorio et al. [63] found no alterations in gangliosides in GD oligodendrocytes. The lack of in vitro models reporting gangliosides in other neural cells such as astrocytes and microglia is a shortcoming towards the current understanding of neuropathology in GD, and whilst brain gangliosides are clearly perturbed in GD, likely contributing to neuropathological outcomes, it is difficult to assess the cause and effects of neurodegeneration in GD without having an understanding of all individual cellular perturbations.

#### Liquid biopsies reflect variable sphingolipid change

From the literature searches, twelve additional studies reported on ceramide and gangliosides from GD patient liquid biopsies, predominantly blood, blood-products, and urine (Supp. Table 4). Plasma sphingolipids have been reported in 132 individuals from seven separate reports wherein G_M3_ was elevated in 70 individuals (100%) and DHC was decreased in 60 patients (57%) [64, 65]. THC was also either unchanged or reduced (69%) [21, 66, 67], and ceramide was unchanged [21, 68, 69], reduced [65], or increased [70] depending on the individual isoform. For example, Meikle et al. [65] found significantly reduced C16:0 ceramide in plasma from 30 type 1 GD patients using mass spectrometry, whereas Byeon and colleagues [70] reported elevated C16:0 ceramide in 3 different type 1 GD patients using similar quantification techniques. Other biopsies of serum, dried blood spots, urine, cerebral spinal fluid, erythrocytes, leukocytes, lung, and bone marrow (*n* = 96 patients and two in vivo mouse models) and a human embryonic kidney cell model (*n* = 3) showed unchanged or variable levels of ceramide and DHC which was also dependent on the specific acyl chain length (Supp. Table 4) [31, 66, 68, 70–77]. The differences in sphingolipids harbouring varying acyl chain lengths are likely reflective of a multitude of factors, including the intrinsic needs of cells for sphingolipid structures and their subsequent release into the surrounding environment, the time at which measurements are taken, individual sphingolipid metabolic/turnover rates, and distinct biologic variability between individuals. Plasma/liquid biopsies are, however, capable of detecting consistent GlcCer and GluSph accumulation and serve as reliable tools for biomarker analysis, diagnosis, and treatment response [69, 78]; the inconsistency of secondary sphingolipid reporting, particularly for ceramide and DHC, suggests that their use as individual tissue/cell surrogate models may not be appropriate.

**Fig. 4:**
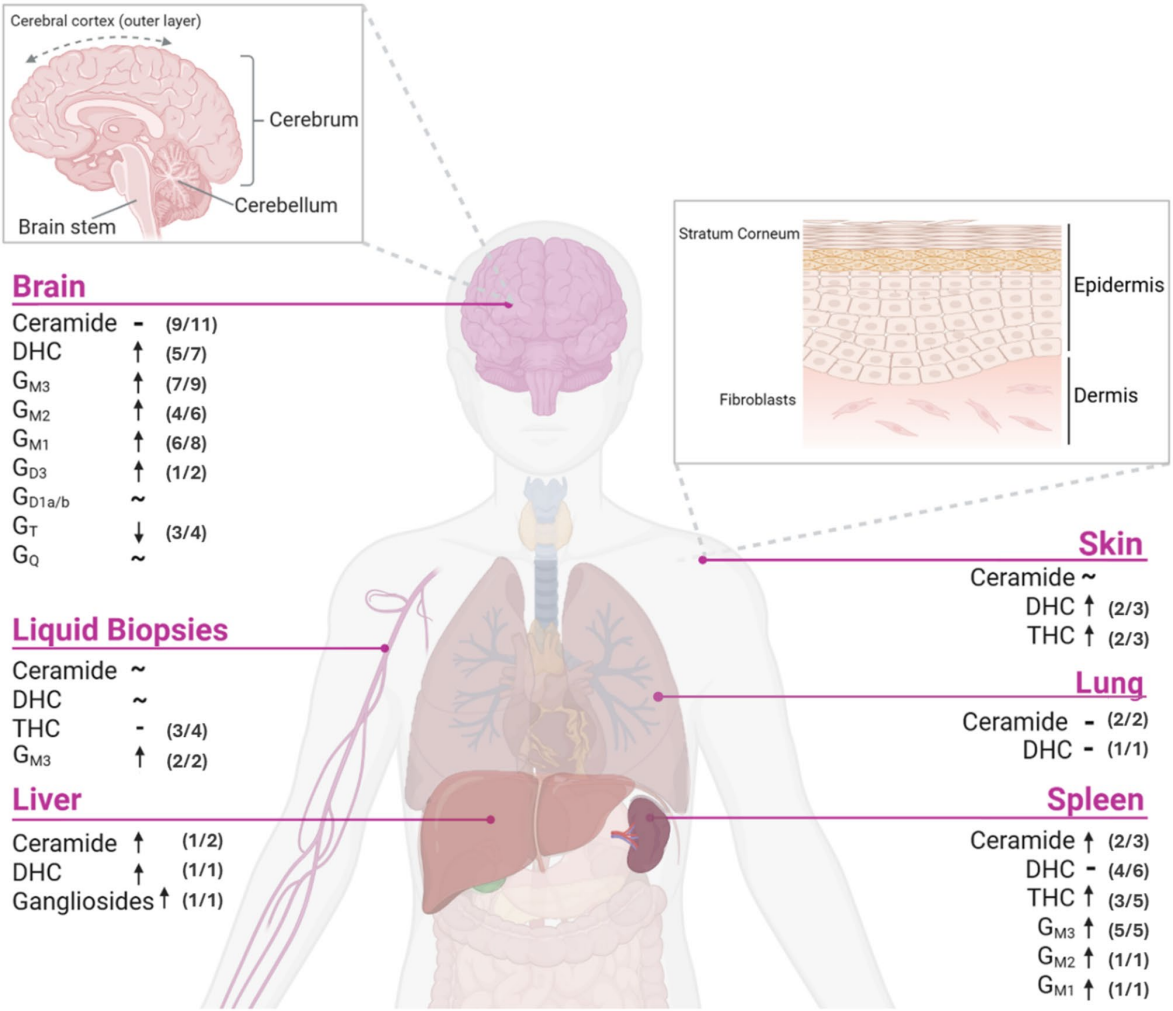
Sphingolipids in Gaucher disease. Changes in ceramide, dihexosylceramide (DHC), trihexosylceramide (THC), and G_M3_, G_M2_, G_M1_, G_D3_, G_D2_, G_D1a/b_, G_T_, and G_Q_ gangliosides, from 49 different studies were grouped by tissue and reflected as either increased (upward arrow), decreased (downward arrow), unchanged (dash symbol, – ), or variable (tilde symbol, ~) compared to healthy controls. *The proportion of studies with determined change (increase*,* decrease*,* unchanged)*,* relative to total reports*,* is listed in parentheses for each sphingolipid and each organ/tissue.* Liquid biopsies encompass reports of blood/blood products, and/or urine. Image created using BioRender.com

## Discussion

###  Compensation of ceramide in GD: friend or foe?

There is logical expectation that ceramide should be reduced in GD, following the loss of its generation from GlcCer, but there is discordance in reports of ceramide across GD cells and tissues. A portion of the inconsistency can be attributed to biological/genetic variability inherent in GD, and the type and efficacy of the lipid extraction and quantification techniques employed. For example, from the 54 reports included in this study, there were seven different quantification techniques used to measure sphingolipids in GD cells and tissues, with mass spectrometry (n = 22) and TLC (n = 22) the most common. With the exception of two reports, most studies that utilised TLC were > 20 years old, which is unsurprising since the introduction of mass spectrometry [79]. Even with mass spectrometry it can be challenging to determine sphingolipid alterations as instrument platforms and acquisition parameters typically vary. Quantification practices also differ (e.g. internal standards vs. standard curves), and data are often normalised to different units (e.g. protein, sample volume, cell count). While standardising all methodological variables across studies is impractical, transparent reporting of analytical conditions and the inclusion of appropriate controls is critical to enable robust and meaningful data comparison. It is also noteworthy that a few studies compiled here reflect atypical GD caused by deficiencies of the Sap C domain in *PSAP* or its cleaved encoded protein, a cofactor of GCase that is necessary for complete hydrolysis of GlcCer [40]. These data should be interpreted separately from classical GCase-GD, as Sap C-deficiencies can also impact the catalytic potential of other lysosomal hydrolases such as acid ceramidase, galactosylceramidase, and acid sphingomyelinase [19]. Spleen and liver ceramide appear to be elevated, whereas brain ceramide is largely unchanged, and skin ceramide varies case-by-case. An in vitro model of GD, in which monocyte-derived macrophage GCase is irreversibly chemically-inhibited with CBE, revealed decreased ceramide at early stages in culture, but normalised with time underscoring the cells’ drive to restore ceramide homeostasis [30]. We also recently demonstrated an upregulation of sphingolipid de novo synthesis in the same macrophage model, wherein stable isotope labelling revealed increased incorporation of palmitate into ceramide and other sphingolipids, which we proposed to be a compensatory mechanism to correct for the catabolic defect in ceramide production [80]. Whether this mechanism is also employed by other cells, contributing to normal ceramide concentrations in the brain or varied ceramide concentrations in the skin, remains to be seen.

Ceramide has been implicated in a number of subcellular processes including regulation of cell death, proliferation, and inflammatory processing [66]. Previously, an increase in the synthesis of de novo C16:0 ceramide has been linked to aberrant inflammasome activation and a pro-inflammatory environment [81, 82]. de novo synthesised C16:0 ceramide is suggested to act as a key component of signalling cascades that lead to perturbed inflammatory processes and insulin resistance [83, 84]. Given that pro-inflammatory hypercytokinemias are associated with sphingolipid-laden macrophages in GD [85–89], it is not unreasonable to suggest that the de novo-driven elevation of ceramide in GD cells might contribute to this chronic proinflammatory phenotype. Long chain ceramides have also been implicated as cell death regulators, acting as secondary messengers in both intrinsic and extrinsic apoptotic pathways [90, 91], whereas very-long chain ceramides exhibit pro-survival functions, playing roles in cell proliferation and growth [91, 92]. In a recent study that explored apoptotic function in GD patients with a dysregulated immune phenotype, Fas-induced apoptosis and caspase activation were defective in 70% of subjects [93]. Another study, which analysed the gene expression profiles of 20 type 1 GD patients, found that genes related to apoptosis, including *CASP*, *NFкβ*, and *BCl2* were all upregulated compared to the control cohort [94]. In this same report, *NTRK1*, relating to neuronal survival, was also significantly decreased, and *BNC1* and *IL1β* genes, implicated in neurodegeneration and apoptosis, were stimulated, indicating dysregulation of intrinsic cell death pathways and impaired cell survival [94]. Other neurological conditions such as Parkinson’s disease, Alzheimer’s disease, Huntington’s disease, and multiple sclerosis, which highlight aberrations of apoptotic events and reduced cell survival, also report an imbalance of ceramide as a common feature [95]. This is contrary to the lack of ceramide change seen in the GD brain, which could suggest the activation of compensatory ceramide mechanisms contributing to the downstream signalling pathologies, opposed to the production of ceramide itself. It is therefore worth investigating whether increased de novo ceramide production is also employed in GD neuronal cells.

Aside from upregulation of de novo synthesis, activation of the salvage and/or sphingomyelinase pathways may also contribute to the cell’s reaction to the loss of ceramide from GlcCer catabolism. Increased SM hydrolysis may be a key response in GD since SM comprises 50–80% of total sphingolipids, depending on the cell/tissue type, and is therefore readily available for degradation [79, 96]. Whilst ceramide recovery may be beneficial to the cell, the expense of SM, particularly at the PM where it is primarily localised, may have consequences on intra- and extracellular signalling. SM is a regulator of membrane stabilisation, receptor clustering, growth signalling, and vesicular trafficking [96], and alterations are likely to influence these downstream events. For similar reasons, an upregulation of the salvage pathway (sphingosine recycling) in response to the loss of ceramide is likely to have consequences for cellular signalling. The conversion of accumulated GluSph to sphingosine by neutral GCase (Fig. 2) may serve to regulate sphingosine and ceramide if increased salvage pathway activity were to occur. It is likely that different cells vary in their ceramide homeostatic response, dependent on substrate load, the need for GlcCer turnover/ceramide, and the availability of other sphingolipids for recycling.

### Elevated DHC and neuroinflammation in GD

All but one of the studies included herein reported levels of sphingolipids in whole/regional brain or neuronal in vitro models (the exception being an oligodendrocyte report [63]). No other glial cell has been analysed, including astrocytes or microglia (resident ‘macrophage of the brain’), and the current understanding of neuroinflammatory pathologies in GD is limited to whole brain or sub-regional analyses. That said, increased expression of glial fibrillary acid protein and other neuroinflammatory markers such as interleukin-1β, tumour necrosis factor-α, and type 1 interferon are often noted in the GD brain [97, 98], all markers of neuroinflammation employed by glial cells [99]. It can be hypothesised that sphingolipid changes within microglia and astrocytes might initiate an inflammatory cascade and the release of these proinflammatory cytokines. A candidate for the induction of these responses is elevated DHC, which was found to be consistent across whole or sub-regional brain analyses from GD patients and in vivo models (Fig. 4, Supp. Table 4). However, with such limited reporting of sphingolipid concentrations in the brain from types 1 and 3 GD, it is difficult to assess whether DHC might correlate with neuronopathic outcomes akin to the neuroinflammatory markers. One report found DHC marginally higher in the cerebral cortex in acute neuronopathic forms compared to chronic neuronopathic GD and controls [27], which could indicate a correlation between DHC accumulation and GD phenotype/increased neuroinflammation. In fact, elevated DHC is associated with microgliosis, astrogliosis, and the production of inflammatory mediators such as cytokines, chemokines, and reactive oxygen species [100–102]. But the question remains as to whether the individual intracellular sphingolipid perturbation comes first, or each of the reactive astrogliosis, microglial activation, and neuronophagia phenotypes are responses to dysfunction from one or more neighbouring cells. Microglia are a good candidate for the induction of the neuroinflammatory cycle because, like macrophages, they are burdened by extracellular lipids through their phagocytic acquisition of apoptotic and senescent circulating cells [30, 34]. The uptake of lipids in GD macrophages from surrounding cells has previously resulted in enhanced secretion of pro-inflammatory cytokines [103] and it is likely that a similar mechanism would ensue in microglia. Whether microglia spark the neuroinflammatory cycle with this added storage burden, or whether there are intracellular sphingolipid changes in other neuronal and glial cells that fire the release of pro-inflammatory mediators, triggering microglial activation, is unknown. Future work should seek to clarify the role of DHC and other sphingolipids in individual GD cells as a potential trigger for neuroinflammatory signalling.

### Altered gangliosides and potential neurodevelopmental consequences in GD

Although present in all tissues, gangliosides predominate in the brain, accounting for 80% of all brain glycans, and are differentiated from other sphingolipids by the presence of a sialic acid [4]. Comprising > 200 individual species due to the variation in ceramide base composition with acyl chains of different degrees of saturation and length [104], temporal ganglioside expression is critical for proper neurodevelopment [105]. For example, simple gangliosides, G_M3_ and G_D3_, correlate with early embryogenic events of neuronal proliferation, differentiation, and modulation of growth factor signalling, and their prevalence dramatically reduces postnatally [106, 107]. Complex gangliosides however, predominate post-maturation with G_D1a_, G_D1b_, G_M1_, and G_T1b_ accounting for > 90% in the fully developed brain [108]. Inasmuch, complex gangliosides function as key regulators of neural signalling events, including synaptic plasticity, axonal growth, and myelination [109]. Complex G_T_, G_D1,_ and G_Q_ gangliosides were reportedly decreased in the GD brain, which is opposite to the concurrent reported elevation of their simple ganglioside precursors, G_M3_, G_M2_, G_M1_, G_D3_, and G_D2_ (Fig. 4; Supp. Table 4). The over-abundance of simple gangliosides, which predominate in early neurodevelopment, along with decreased G_D1_ and G_T1_, is suggestive of an immature or underdeveloped brain. In fact, previous reports have implicated impaired neurogenesis as a pathological mechanism in GD. One study highlighted decreased expression of *LMX1B* and *NURR1* neuronal differentiation transcription factors [110], whilst another found a downregulation of canonical Wnt/β-catenin signalling in neuronal precursor cells that resulted in impaired dopaminergic neuronal differentiation [111]. G_M3_ has also been shown to modulate epidermal growth factor receptor via competitive autophosphorylation inhibition, preventing cell proliferation and growth [112]. It would follow that increased G_M3_ might contribute to a disrupted proliferative phenotype, leading to the profound neuronal loss and neuronophagia pathologies observed in the GD brain [97, 113, 114]. Similarly, the underdeveloped status in GD might result in impaired processes occurring in the mature brain such as regulation of synaptic plasticity and myelination caused by the loss of complex regulatory gangliosides. A number of studies report dysregulated synaptic function and impaired myelination as a pathological feature of GD; one report of murine *Gba* deficiency and chemically-induced murine GCase inhibition found glial activation within nigrostriatal pathways, noting reduced dopamine release, a decrease in post-synaptic density size, and an altered microRNA profile, serving as evidence of synaptic dysfunction [115]. Another study found that loss of GCase in oligodendrocytes inhibited myelination in vitro and resulted in demyelination, axonal degeneration, and astrogliosis neurodegenerative markers in vivo [63]. Gregorio et al. [63] also reported DHC trending upwards in CBE-induced Oli-Neu cells, but did not measure or report any other sphingolipid/ganglioside changes that might link the observed myelination defects. Together, findings here suggest that the common ganglioside changes in GD reflect a pattern of delayed neurodevelopment; the link between ganglioside dysregulation, downstream signalling defects, and the resulting neuropathologies however, is an area that requires further investigation.

### Altered gangliosides as a potential neuroprotective response in GD

Excess ganglioside has been regarded as a potential cause of cell dysfunction, however, some individual ganglioside species have been positively associated with neural regeneration [116]. It has not yet been considered whether GD neural cells could action a compensatory elevation of neuroprotective gangliosides in response to cellular distress. For example, G_M1_ is positively associated with regeneration of the nervous system in adults and has been advocated as a potential therapeutic for central nervous system disease and injury [117]. Exogenous G_M1_ administration has been shown to alleviate neuropathological features such as impaired apoptosis, α-synuclein aggregation, and neurotoxicity in neurodegenerative disorders and models of neuronal damage [116–118]. Although it is evident that G_M1_ is elevated in the GD brain, and that G_D_ and G_T_ species are reduced, there is a lack of clarification about whether these changes are indeed concomitant and employed as a compensatory neuroprotective response. The lack of time-dependent studies is a key limitation toward understanding the role of ganglioside alterations in GD as all measurements heretofore have been a snapshot in time rather than following the trajectory of brain development in real-time. Without an understanding of how gangliosides change over time, the dynamics of sphingolipid metabolism are lost, and the mechanisms of disease progression cannot be elucidated.

## Conclusions

In summary, whilst secondary sphingolipid changes are common in GD, clarification of individual cellular perturbations and their contribution to disease pathology is lacking. Reports of sphingolipid aberrations are generally consistent across different patient-derived samples and models, with most reports noting elevated DHC, G_M1_, G_M2_, G_M3_, G_D2_, and G_D3_ gangliosides. G_T_ gangliosides on the other hand, are typically reduced, and G_D1_ and G_Q_ gangliosides are sometimes elevated and sometimes reduced, depending on the cell/tissue analysed. Ceramide was also inconsistently reported to be elevated, unchanged, or decreased across 33 individual reports and was generally tissue specific: spleen and liver ceramide was mostly elevated in GD, brain ceramide was not different, and skin ceramide was highly variable. These changes could be due to homeostatic ceramide responses with varying regulatory success in select GD cells/tissues that cannot be fully captured without time course analyses, consistent methodologies, and individual cell examination. Since different cells have varying requirements for GlcCer, how each cell copes with the storage burden, and how these changes contribute to tissue pathology are also likely variable.

## Supplementary Information

Below is the link to the electronic supplementary material.

Supplementary Material 1.

## Data Availability

Not applicable.

## Abbreviations

GD: Gaucher disease
GlcCer: Glucosylceramide
GluSph: Glucosylsphingosine
GCase: Acid beta glucocerebrosidase
DHC: Dihexosylceramide
THC: Trihexosylceramide
ER: Endoplasmic reticulum
PM: Plasma membrane
CERT: Ceramide transport protein
SM: Sphingomyelin
GalCer: Galactosylceramide
C1P: Ceramide-1-phosphate
S1P: Sphingosine-1-phosphate
SMase: Sphingomyelinase
PSAP: Prosaposin
Sap C: Saposin C
TLC: Thin layer chromatography
CBE: Conduritol B epoxide
