# Supplementary material for: Sphingolipids in Gaucher disease: a systematic review
Source: Orphanet J Rare Dis. 2025 Nov 6;20:565. doi: 10.1186/s13023-025-04015-5 (PMC12590618; doi:10.1186/s13023-025-04015-5)
Supplement: Supplementary file 1 — Supplementary Material 1. [file 13023_2025_4015_MOESM1_ESM.docx]

**Supplemental Material**

**Sphingolipids in Gaucher disease: a systematic review**

Ashleigh Lake^1,2^ and Maria Fuller^1,2,3^

^1^Genetics and Molecular Pathology, SA Pathology at Women’s and Children’s Hospital, North Adelaide 5006, Australia

^2^School of Biological Sciences, University of Adelaide, Adelaide 5000, Australia

^3^Adelaide Medical School, University of Adelaide, Adelaide 5000, Australia

Corresponding author: Professor Maria Fuller, [maria.fuller@adelaide.edu.au](mailto:maria.fuller@adelaide.edu.au), 72 King William Road, North Adelaide 5006, Australia

**Supplementary Table 1:** Quality assessment of non-randomised cohort studies using the Newcastle-Ottawa scoring tool.

|  | Representatives of the exposed cohort | Selection of the non-exposed cohort | Ascertainment of exposure | Outcome of interest not present at start of the study | Comparability of the cohorts based on design of the analysis | Assessment of outcome | Follow-up long enough for outcomes to occur | Adequacy of follow-up of cohorts | Total Score |
| --- | --- | --- | --- | --- | --- | --- | --- | --- | --- |
| Suomi 1965 | * | 0 | 0 | 0 | 0 | 0 | 0 | 0 | 1 |
| Ghauharali van-der Vlugt 2008 | * | 0 | * | 0 | 0 | 0 | 0 | 0 | 2 |
| Nilsson 1985 | 0 | 0 | * | 0 | 0 | 0 | * | * | 3 |
| Nilsson 1982 | * | 0 | * | 0 | 0 | 0 | 0 | 0 | 2 |
| Hulkova 2005 | 0 | 0 | 0 | 0 | 0 | 0 | 0 | 0 | 0 |
| Makita 1966 | 0 | 0 | 0 | 0 | 0 | 0 | 0 | 0 | 0 |
| Kuske and Rosenberg 1972 | 0 | 0 | 0 | 0 | 0 | 0 | 0 | 0 | 0 |
| Sidransky 1996 | * | 0 | * | * | 0 | 0 | 0 | 0 | 3 |
| Dawson 1972 | 0 | 0 | 0 | 0 | 0 | 0 | 0 | 0 | 0 |
| Kubota 1972 | 0 | 0 | 0 | 0 | 0 | 0 | 0 | 0 | 0 |
| Pampols 1999 | 0 | 0 | 0 | 0 | 0 | 0 | 0 | 0 | 0 |
| Sudo 1977 | 0 | 0 | 0 | 0 | 0 | 0 | 0 | 0 | 0 |
| Gonzalez-Sastre 1974 | 0 | 0 | 0 | 0 | 0 | 0 | 0 | 0 | 0 |
| Moraitou 2004 | * | * | 0 | 0 | 0 | 0 | * | 0 | 3 |
| Meikle 2008 | * | * | * | 0 | * | 0 | * | * | 6 |
| Mistry 2014 | 0 | 0 | 0 | 0 | 0 | 0 | 0 | 0 | 0 |
| Byeon 2015 | * | * | 0 | 0 | 0 | 0 | * | * | 4 |
| Whitfield 2002 | * | * | * | 0 | * | 0 | 0 | 0 | 4 |
| Spiewak 2023 | 0 | * | * | 0 | * | 0 | 0 | 0 | 3 |
| Kilbansky 1976 | 0 | 0 | 0 | 0 | 0 | 0 | 0 | 0 | 0 |
| Vance 1969 | 0 | 0 | 0 | 0 | 0 | 0 | 0 | 0 | 0 |
| Groener 2007 | * | * | * | * | 0 | 0 | * | * | 6 |
| Gornati 2002 | 0 | 0 | * | * | 0 | 0 | 0 | 0 | 2 |
| Schiffmann 2023 | * | * | * | * | * | * | * | * | 8 |

**Supplementary Table 2:** Quality assessment of *in vitro* reports using QUIN. The following categories were excluded as not applicable to score calculation: detailed explanation of sample size calculation; operator details; randomisation; outcome assessor details; blinding.

| Reference | Clearly stated aims/objectives | Detailed explanation of sampling technique | Details of comparison group | Detailed explanation of methodology | Method of measurement outcome | Statistical analysis | Presentation of results | Final Score | Risk |
| --- | --- | --- | --- | --- | --- | --- | --- | --- | --- |
| Hein 2007 | 2 | 2 | 2 | 2 | 1 | 2 | 2 | 92.9 | Low |
| Hattersley 2013 | 2 | 1 | 2 | 2 | 2 | 2 | 2 | 92.9 | Low |
| Hein 2008 | 2 | 2 | 2 | 2 | 2 | 2 | 2 | 100.0 | Low |
| Hein 2017 | 2 | 1 | 2 | 2 | 2 | 2 | 2 | 92.9 | Low |
| Vaccaro 2010 | 2 | 2 | 1 | 2 | 2 | 2 | 2 | 92.9 | Low |
| Fuller 2008 | 2 | 1 | 1 | 2 | 2 | 2 | 2 | 85.7 | Low |
| Schonauer 2017 | 2 | 2 | 2 | 2 | 2 | 2 | 2 | 100.0 | Low |
| Tatti 2015 | 2 | 2 | 1 | 2 | 2 | 2 | 2 | 92.9 | Low |
| Ceni 2024 | 2 | 1 | 1 | 2 | 2 | 2 | 2 | 85.7 | Low |
| Saito and Rosenberg 1985 | 2 | 1 | 1 | 2 | 2 | 1 | 1 | 71.4 | Low |
| Barton and Rosenberg 1975 | 2 | 1 | 1 | 2 | 2 | 1 | 1 | 71.4 | Low |
| Gregorio 2024 | 2 | 2 | 2 | 2 | 2 | 2 | 2 | 100.0 | Low |
| Mazzulli 2011 | 2 | 2 | 2 | 2 | 2 | 2 | 2 | 100.0 | Low |
| Korkotian 1999 | 2 | 1 | 1 | 2 | 2 | 1 | 1 | 71.4 | Low |
| Magalhaes 2016 | 2 | 2 | 2 | 2 | 2 | 2 | 2 | 100.0 | Low |
| Lunghi 2022 | 2 | 1 | 1 | 2 | 2 | 2 | 2 | 85.7 | Low |
| Campeau 2009 | 2 | 1 | 1 | 1 | 1 | 2 | 2 | 71.4 | Low |
| Kim 2018 | 2 | 2 | 2 | 2 | 2 | 2 | 2 | 100.0 | Low |

**Supplementary Table 3:** Quality assessment of *in vivo* studies using SYRCLE.

| Reference | Allocation sequence | Groups similar at baseline | Concealment | Random housing | Investigators blinded | Random assessment | Outcome assessor blinded | Incomplete data | Free of selective outcome reporting | Free of other problems |
| --- | --- | --- | --- | --- | --- | --- | --- | --- | --- | --- |
| Karageorgos 2016 | Unclear | Low | Unclear | Low | Unclear | High | High | Low | Low | Low |
| Stephens 1979 | Unclear | Low | Unclear | Unclear | Unclear | Unclear | Unclear | Low | Low | Low |
| Holleran 1994 | Unclear | Low | Unclear | Unclear | Unclear | Unclear | Unclear | Low | Low | High |
| Doering 1999 | Unclear | Low | Unclear | Unclear | Unclear | Unclear | Unclear | Low | Low | Low |
| Liou 2019 | Unclear | Low | Unclear | Low | Unclear | Unclear | Unclear | Low | Low | Low |
| Sun 2010 | Unclear | Low | Unclear | Low | Unclear | Unclear | Unclear | Low | Low | Low |
| Duffy 2024 | Unclear | Low | Unclear | Unclear | Unclear | High | Unclear | Low | Low | Low |
| Gregorio 2024 | Unclear | Low | Unclear | Unclear | Low | High | Unclear | Low | Low | Low |
| Farfel-Becker 2014 | Unclear | Low | Unclear | Unclear | Unclear | Unclear | Unclear | Low | Low | Low |
| Smith 2018 | Unclear | Low | Unclear | Low | Unclear | Unclear | Unclear | Low | Low | Low |
| Barnes 2014 | Unclear | Low | Unclear | Unclear | Unclear | Unclear | Unclear | Low | Low | Low |
| Pavlova 2015 | Unclear | Low | Unclear | Unclear | Unclear | Unclear | Unclear | Unclear | Low | Low |

**Supplementary Table 4: Sphingolipids in different GD tissues, cells, and liquid biopsies.**

| Author, year | Sample type | Sample, GD variant (n) | Sphingolipid assay method | Sphingolipid level in GD cohort | Key finding |
| --- | --- | --- | --- | --- | --- |
| *Spleen* | | | | | |
| Suomi *et al.* 1965 | Biopsy | Spleen, unknown variant (n=8) | TLC | Ceramide: 0.88 mg/g  DHC:  Patient 1: 0.74 mg/g  Patient 2: 0.68 mg/g  Patient 3: 0.79 mg/g  Patient 4: 0.58 mg/g  Patient 5: 0.37 mg/g  Patient 6: 0.84 mg/g  Patient 7: 0.79 mg/g  Patient 8: 1.12 mg/g | Ceramide only detected in one patient and one control, two times higher in Gaucher spleen. DHC not different in GD and control spleens. |
| Ghauharali-van der Vlugt *et al.* 2008 | Biopsy | Spleen, type 1 (n=4) | HPLC | Ceramide: 0.25 mmol/kg wet weight  THC: 0.02 mmol/kg wet weight  G_M3_: 0.15 mmol/kg wet weight | Ceramide 3-fold higher compared to controls. THC unchanged. G_M3_ 5-fold higher compared to controls. |
| Nilsson *et al.* 1985 | Autopsy | Spleen, type 1 (n=2) | TLC and densitometry | Patient 1: 1.65 A.U.  Patient 2: 0.95 A.U. | Elevated gangliosides in GD spleen. |
| Nilsson *et al.* 1982 | Autopsy | Spleen, type 1 (n=4), type 2 (n=3), and type 3 (n=12) | TLC and GLC | Type 1: 1.31 gangliosides mmol/kg  Type 2: 1.37 gangliosides mmol/kg  Type 3: 1.23 gangliosides mmol/kg | DHC variable, but unchanged in spleen. Elevated gangliosides in spleen (predominantly G_M3_) with no differences between GD type. |
| Hulkova *et al.* 2005 | Biopsy | Spleen, type 1 (n=2) | Immunohistochemistry and TLC and densitometry | 112% and 82% densitometric DHC % relative to mean controls. | Strong decrease/absence of immunodetectable DHC. TLC spleens not significantly different from controls. |
| Makita *et al.* 1966 | Biopsy | Spleen, type 1 (n=1) and type 2 (n=2) | TLC | Patient 1 (type 1): DHC, 0.18 mg/g; THC, 0.06 mg/g; globoside, 0.14 mg/mg  Patient 2 (type 2): DHC, 0.15 mg/g; THC, 0.03 mg/g; globoside, 0.23 mg/g.  Patient 3 (type 2): DHC, 0.6 mg/g | Unchanged GSL content in GD. |
| Kuske and Rosenburg 1972 | Biopsy | Spleen, unspecified variant (n=4) | TLC | DHC: 0.397 mg/g  THC: 0.137 mg/g  Globoside: 0.191 mg/g  G_M3_: 0.198 mg/g | DHC and globoside levels were not significantly different from controls in GD spleens, whereas THC and G_M3_ were significantly elevated. |
| Karageorgos *et al.* 2016 | *In vivo* | Spontaneous GD sheep spleen (n=3) | LC-ESI-MS/MS | DHC: 1.4 nmol/mg  THC: 0.47 nmol/mg | No significant ceramide change. DHC and THC elevated. |
| Stephens *et al.* 1979 | *In vivo* | GD-CBE mouse spleen (n=2) | TLC | DHC: 104 nmol/mg  THC: 58 nmol/mg  Globoside: 123 nmol/mg  G_M3_: 162 nmol/mg | DHC, THC, G_M3_, and globoside were all elevated in GD adult spleen. |
| *Skin (including fibroblasts)* | | | | | |
| Sidransky *et al.* 1996 | Biopsy | Epidermis, type 1 (n=2), type 2 (n=2), type 3 (n=1) | HPTLC and densitometry | Type 2: 2.7 glucosylceramide:ceramide ratio | Decreased ceramide in type 2, but not types 1 or 3. |
| Dawson *et al.* 1972 | Biopsy | Skin fibroblasts, adult GD (n=2) | TLC | G_M3_: 1.79 and 1.47 µmoles/g  G_D3_: 1.21 and 1.23 µmoles/g | No comment on GSL change although G_M3_ is elevated compared to controls. |
| Holleran *et al.* 1994 | *In vivo* | *Gba* null allele in mice, epidermis and stratum corneum (n=≥3) | HPTLC | No quantitation reported. | Ceramide significantly reduced. |
| Doering *et al.* 1999 | *In vivo* | RecNciI GD mice, epidermis (n=4) | TLC and photodensitometry | Up to 40% lower than controls. | Overall reduction of ceramides. |
| Liou *et al.* 2019 | *In vivo* | Epidermis, *Gba* point mutation crossed with Sap C-deficient mice (n=3-6) | LC-ESI-MS/MS | WT/C, 9V:C, and OS:C mice - increased <2-fold. OS mice increased >2-fold. | All Sap C/GCase mice had significantly elevated ceramide. OS *Gba* homozygous mice had elevated ceramide, but 9V homozygous mice had unchanged levels. |
| Vaccaro *et al.* 2010 | *In vitro* | Sap C-deficient fibroblasts, case report | HPTLC and fluorography with immunofluorescence | 4-5 times stronger [^14^C]ceramide bands than controls | Accumulated [^14^C]ceramide following radioactive loading studies. |
| Fuller *et al.* 2008 | *In vitro* | Fibroblasts, type 1 (n=7), type 2 (n=5) | ESI-MS/MS | Ceramide: 2.5 nmol/mg protein at 1 week post-confluence. 7.9 nmol/mg protein at 6 weeks post-confluence.  Type 1: DHC, 2.2 nmol/mg (1 week), 4.7 (6 weeks); THC, 5.7 (1 week), 10 (6 weeks).  Type 2: DHC, 3.6 (1 week), 7.1 (6 weeks); THC, 5.0 (1 week), 8.4 (6 weeks). | Elevated ceramide. DHC and THC significantly elevated compared to controls, but not different between GD types 1 and 2. |
| Schonauer *et al.* 2017 | *In vitro* | GD fibroblasts, type (n=5), type 2 (n=5), type 3 (n=5), unknown variant (n=1) | Q-TOF MS/MS | No quantitation reported. | Ceramide unchanged. |
| Tatti *et al.* 2015 | *In vitro* | Fibroblasts, N370S homozygote (n=1), L444P homozygote (n=1), and Sap C deficiency (n=2) | Immunofluorescence | No quantitation reported. | Ceramide elevated in Sap C fibroblasts (undetected in controls and GD cells). |
| Ceni *et al.* 2024 | *In vitro* | Fibroblasts, type 1 (n=1), type 2 (n=2) | Confocal microscopy | 5-fold increase in L444P homozygous fibroblasts and 110% increase in R131C homozygous fibroblasts. | G_M1_ elevated in severe GD and unchanged in mild (N370S) GD. |
| Saito and Rosenberg 1985 | *In vitro* | Fibroblasts, type 2 (n=3) | HPTLC and densitometry | DHC: 0.54 nmol/mg protein  G_M3_: 4.21 nmol/mg protein  G_M2_: 1.94 nmol/mg protein  G_M1_: 0.19 nmol/mg protein  G_D3_: 0.16 nmol/mg protein  G_D1a_: 0.5 nmol/mg protein  Globoside: 0.82, 1.98 nmol/mg protein | All GSLs within normal range. |
| Barton and Rosenberg 1975 | *In vitro* | Skin fibroblasts, case report unspecified variant | TLC and liquid scintillation spectrometry | DHC: 863.9 activity/min/mg  THC: 200.9 activity/min/mg  Globoside: 154.1 activity/min/mg | Significant levels of activity of DHC and Globoside greater than controls. |
| *Brain* |  |  |  |  |  |
| Nilsson *et al.* 1985 | Autopsy | Brain type 1 (n=2) | TLC and densitometry | Total gangliosides: Patient 1: cerebral cortex, 3.51 nmol/kg; cerebellar cortex, 3.53 nmol/kg  Patient 2: cerebral cortex, 3.44 nmol/kg; cerebellar cortex, 2.96 nmol/kg | Gangliosides within normal range in GD brain. |
| Kubota *et al.* 1972 | Autopsy | Cerebrum, type 2 case report | TLC AND GLC | No quantitation reported. | Elevated G_M3_ ganglioside. |
| Pampols *et al.* 1999 | Autopsy | Grey and white brain matter, Sap C-deficiency case report | TLC and densitometry | Grey matter: G_T1_, 5 µmol/g; G_D1b_, 12 µmol/g; G_D1a_, 16 µmol/g; G_M1_, 65 µmol/g  White matter: G_T1_, 6 µmol/g; G_D1b_, 12 µmol/g; G_D1a_, 24 µmol/g; G_M1_, 56 µmol/g | Minor reductions of G_T_ gangliosides and elevations of G_M1_ ganglioside but overall, statistically insignificant. |
| Nilsson and Svennerholm 1982 | Autopsy | Cerebral cortex, type 2 (n=5), type 3 (n=8), atypical type 1/3 (n=1) and cerebellar cortex, type 2 (n=4), type 3 (n=6), atypical type 1/3 (n=1) | HPTLC | DHC (cerebral cortex \| cerebellar cortex):  Patient 1 (type 2): 56\|102  Patient 2 (type 2): 62\|140  Patient 3 (type 2): 130\|160  Patient 4 (type 2): 56\|N/A  Patient 5 (type 2): 38\|96  Patient 6 (type 3): 32\|N/A  Patient 7 (type 3): 26\|120  Patient 8 (type 3): 78\|82  Patient 9 (type 3): 55\|78  Patient 10 (type 3): 53\|N/A  Patient 11 (type 3): 44\|139  Patient 12 (type 3): 35\|160  Patient 13 (type 3): 115\|201  Patient 14 (type 1/3): 39\| 128  Gangliosides (cerebral cortex \| cerebellar cortex):  Patient 1 (type 2): 3.69\|2.93  Patient 2 (type 2): 2.53\|2.73  Patient 3 (type 2): 3.2\|2.98  Patient 4 (type 2): 3.5\|N/A  Patient 5 (type 2): 3.0\|2.77  Patient 6 (type 3): 3.02\|N/A  Patient 7 (type 3): 3.45\|3.02  Patient 8 (type 3): 2.43\|2.87  Patient 9 (type 3): 3.37\|3.11  Patient 10 (type 3): 3.13\|N/A  Patient 11 (type 3): 3.22\|2.66  Patient 12 (type 3): 3.61\|3.24  Patient 13 (type 3): 3.65\|2.73  Patient 14 (type 1/3): 3.2\|3.32 | DHC was slightly elevated in the cerebral cortex of type 2 cases and moderately elevated in cerebellar cortex in all GD patients. Distribution of gangliosides was within normal ranges in type 3 cases, whereas G_M3_ and G_M2_ were significantly elevated in type 2 brains. THC and tetraglycosylceramide were increased in all cases. |
| Sudo *et al.* 1977 | Autopsy | Brain (grey matter), type 2 case report | GLC | DHC: 0.11 mg/g | Slightly higher DHC in GD. |
| Gonzalez-sastre *et al.* 1974 | Autopsy | Brain, type 2 case report | TLC and densitometry | Frontal lobe: G_T_, 11%; G_D1b_, 10%; G_D1a_, 44.3%; G_M1_, 22.9%; G_M2_, 8.7%; G_M3_, 3.1% Temporal lobe: G_T_, 9.5%; G_D1b_, 9.3%; G_D1a_, 49.6%; G_M1_, 22.7%; G_M2_, 6.0%; G_M3_, 2.9%  Occipital lobe: G_T_, 13.3%; G_D1b_, 14.5%; G_D1a_, 46.2%; G_M1_, 17.6%; G_M2_, 5.8%; G_M3_, 2.6% Cerebellum: G_T_, 22.8%; G_D1b_, 10.6%; G_D1a_, 23.1%; G_M1_, 1.4%; G_M2_, 4.7%; G_M3_, 3.3% | G_M3_ was undetected in the control and increased in the GD case. G_M1_ was also elevated. The proportion of G_T_ decreased whereas G_D1b_ increased. |
| Stephens *et al.* 1979 | *In vivo* | GD-CBE mouse, adult brain (n=9) and young brain (n=5) | TLC | Adult: G_M2_, 0.99%; G_M1_: 13.6%; G_D1a_, 31.4%; G_D1b_/G_T1_, 49.4%  Young: G_M3_: 1.0%; G_M2_, 3.0%; G_M1_, 6.4%; G_D1a_, 50.8%; G_D1b_, 13.3%; G_T1_, 26.8% | G_M1_ was significantly elevated and G_D1b_/G_T_ was significantly reduced in adult GD brain. Only G_M3_ was significantly elevated in young GD brain. |
| Sun *et al.* 2010 | *In vivo* | Combined Sap C/GCase point mutation, mouse brain (n=3) | LC-MS | 1.9-fold higher DHC than controls. | Ceramide unchanged. DHC significantly elevated in cerebellum but not spinal cord. |
| Karageorgos *et al.* 2016 | *In vivo* | Spontaneous GD sheep brain (n=3) | LC-ESI-MS/MS | DHC in hippocampus: 1.6 nmol/mg  DHC in midbrain: 0.97 nmol/mg | Significantly elevated G_M3_, G_M2_, G_M1_ in all brain regions, most prominent in the hippocampus. Marginally elevated DHC in some brain regions. Ceramide unchanged. |
| Farfel-Becker *et al.* 2014 | *In vivo* | GD mouse (Gba^flox/flox^; nestin-Cre) brain, GCase deficiency restricted to neurons and macroglia (n=4-5) | LC-ESI-MS/MS | No quantitation reported. | DHC moderately elevated in caudate putamen and cortex at 14 days. Unchanged in other regions. Total ceramide unchanged in all regions. |
| Duffy *et al.* 2024 | *In vivo* | Gbafl/fl; Tmem119-CreERT2 (microglia-specific *Gba* KO) mice and Gbafl/fl; Thy1-CreERT2,-EYFP (neuron-specific *Gba* KO) mice, brain (n=5) | HPLC-MS/MS | No quantitation reported. | Elevated C18:0 ceramide and reduced C24:1 ceramide at 13-14 weeks, but unchanged ceramide at 16 weeks in neuron-specific GD mice. Slightly reduced C18:0 and C24:1 in microglial-specific GD mice. Total ceramide unchanged in both mouse models. |
| Smith *et al.* 2018 | *In vivo* | GD mouse cortex and sub-cortex, GCase deficiency in all tissues except skin (n=3) | LC-ESI-MS/MS | No quantitation reported. | DHC, G_M3_, G_M2_, G_M1_, G_D2_, G_D3_, and G_D1a_ (C22:0 and C16:0) all significantly elevated in cortex and sub-cortex of GD mice. |
| *Liquid Biopsies* | | | | | |
| Meikle *et al.* 2008 | Biopsy | Plasma, type 1 (n=30) | ESI-MS/MS | Ceramide: C16:0, 0.24 µM; C24:0, 1.6 µM, C24:1 - 1.69 µM  DHC: C16:0, 3.4 µM; C24:0, 0.35 µM; C24:1, 0.71 µM.  THC: C16:0, 0.31 µM; C24:0, 0.07 µM; C24:1, 0.09 µM.  G_M3_: C16:0, 5.2 µM; C24:1. 1.4 µM | Significantly reduced C16:0, C24:0, and C24:1 ceramide. Elevated C24:1 G_M3_. Reduced levels of C16:0 DHC and C16:0, C24:0, C24:1 THC. |
| Mistry *et al.* 2014 | Biopsy | Serum, type 1 (n=41) | HPLC | 1.20 (A.U.) | Ceramide remained within normal control levels. |
| Byeon *et al.* 2015 | Biopsy | Plasma and urine, type 1 (n=3) | nLC-ESI-MS/MS | Ceramide: C16:0 - 2.46 ratio of average peak area and C18:0 - 2.54 in plasma. C24:0 - 2.48 in urine. Greater than 2-fold elevation.  Plasma DHC: 0.9 ratio of average peak area  Urine DHC: 3.46 ratio of average peak area | Elevated C16:0, C18:0 and unchanged C24:0 ceramide in plasma. Unchanged C16:0, C18:0 and elevated C24:0 ceramide in urine. Elevated C24:0 DHC in urine but not plasma. Other DHC isoforms unchanged. |
| Ghauharali-van der Vlugt *et al.* 2008 | Biopsy | Plasma, type 1 (n=40) | HPLC | Ceramide: 9.8 µmol/L.  THC: 1.7 µmol/L  G_M3_: 10.2 µmol/L | Unchanged ceramide. THC unchanged. G_M3_ 3-fold higher in GD. |
| Whitfield *et al.* 2002 | Biopsy | Plasma, mixed cohort (n=30) | ESI-MS/MS | 4.8 µmol/L | Decreased DHC compared to controls. |
| Spiewak *et al.* 2023 | Biopsy | Dried blood spot, type 1 (n=2), type 3 (n=2) | UPLC-MS/MS | No quantitation reported. | DHC and globosides unchanged. |
| Vance *et al.* 1969 | Biopsy | Plasma type (n=2) | TLC and GLC | Plasma DHC, 0.48 µmol/100 mL (patient 1); 0.56 µmol/100 mL  (patient 2); THC, 0.2 µmol/100 mL (patient 1); 0.2 µmol/100 mL (patient 2) | DHC and THC concentrations within the normal range. |
| Hulkova *et al.* 2005 | Biopsy | Bone marrow, type 1 (n=1) | Immunohistochemistry and TLC | No quantitation reported. | Strong decrease/absence of immunodetectable DHC. |
| Groener *et al.* 2007 | Biopsy | Plasma, type 1 (n=11 splenectomised); (n=16 non-splenectomised) | HPLC | 7.2 nmol/mg | Ceramide unchanged. Groups were not separated. |
| Gornati *et al.*  2002 | Biopsy | CSF, type 2 case report | TLC | No quantitation reported. | Traces of DHC in CSF, whereas undetected in controls. |
| Schiffmann *et al.* 2023 | Biopsy | CSF, type 3 (n=9) | LC-ESI-MS/MS | No quantitation reported. | Ceramide in reference ranges at baseline. |
| Pavlova *et al.* 2015 | *In vivo* | Plasma, GD mouse: *Gba*^tm1Karl/tm1Karl^Tg(Mx1-Cre)ICgn/O (n=unknown) | HPLC | No quantitation reported. | Ceramide unchanged. |
| *Liver* |  |  |  |  |  |
| Nilsson *et al.* 1985 | Autopsy | Liver, type 1 (n=2) | TLC and densitometry | Patient 1: 0.78 nmol/kg gangliosides  Patient 2: 0.61 nmol/kg gangliosides | Elevated gangliosides in liver. |
| Sun *et al.* 2010 | *In vivo* | Combined Sap C/GCase point mutation, mouse liver (n=3) | LC-MS | 1.6-fold higher ceramide. | Elevated ceramide. |
| Karageorgos *et al.* 2016 | *In vivo* | GD sheep liver (n=3) | LC-ESI-MS/MS | No quantitation reported. | Ceramide unchanged. DHC elevated. |
| *Lung* |  |  |  |  |  |
| Sun *et al.* 2010 | *In vivo* | Combined Sap C/GCase point mutation, mouse lung (n=3) | LC-MS | No quantitation reported. | Unchanged ceramide. |
| Barnes *et al.* 2014 | *In vivo* | GD mouse: 9V & 9V/null (n=unknown), lung | ESI-LC-MS/MS | No quantitation reported. | Ceramide unchanged. DHC trending upwards but unchanged. |
| *Cells* |  |  |  |  |  |
| Moraitou *et al.* 2014 | Biopsy | Erythrocytes, type 1 (n=24) and 2 (n=3) | HPLC | 68-364 pmol/10^8^ cells (300 pmol/10^8^ cells: median) | Ceramide 3.4-fold decreased in one individual but not significantly different from controls in overall cohort. No inferred differences between GD subtypes with small sample size. |
| Kilbansky *et al.* 1976 | Biopsy | Leukocytes, type 1 (n=10) | TLC | DHC: 94.4 nmol/10^8^ cells. | Elevated DHC in all but 1 patient. |
| Vance *et al.* 1969 | Biopsy | Erythrocytes, two type 1 case reports | TLC and GLC | Erythrocytes: DHC, 1.12 µmol/100 mL; 3.92 µmol/100 mL (patient 2); THC: 1.4 µmol/100 mL (patient 1); 0.84 µmol/100 mL (patient 2) | DHC and THC concentrations within the normal range. |
| Kim *et al.* 2018 | *In vitro* | *GBA1* insertion variant in HEK293-FT cells (n=3) | LC-MS/MS | 0.4-fold decrease of C18:0 ceramide. | C18:1 and C24:1 ceramide significantly decreased in *GBA1* KO Hek293-FT cells. Other isoforms unchanged. |
| Hein *et al.* 2007 | *In vitro* | CBE-THP-1 macrophages (n=3) | ESI-MS/MS | No quantitation reported. | Decreased ceramide at early stages in culture, unchanged with prolonged time. Ceramide elevated at the endoplasmic reticulum. DHC significantly elevated at all times. THC significantly elevated at 2 and 5 days of CBE treatment. |
| Hein *et al.* 2008 | *In vitro* | Membrane microdomains from CBE-macrophages (n=3) | ESI-MS/MS | 1.9-fold increase of ceramide in detergent-resistant membranes. 2.5-fold and 1.7-fold elevation of DHC and THC, respectively. | Ceramide elevated in lipid rafts and unchanged in soluble membranes. Lysosomal lipid rafts exhibit secondary DHC, and THC accumulation. |
| Hattersley *et al.* 2013 | *In vitro* | Conditional *Gba* KO mouse spleen microdomains (n=3) | LC-ESI-MS/MS | No quantitation reported. | Elevated ceramide. Significantly elevated DHC and THC in membrane microdomains from mouse spleen. |
| Hein *et al.* 2017 | *In vitro* | GD sheep, microdomains from spleen (n=3) | LC-ESI-MS/MS | G_M3_, G_M2_, and G_M1_: <0.3 nmol/mg protein | Elevated G_M3_, G_M2_, and G_M1_. |
| *Hein *et al.* 2017 | *In vitro* | Microdomains from GD sheep brain (n=3) | LC-ESI-MS/MS | Combined G_M3_, G_M2_, G_M1_: 65.4 nmol/mg | Unchanged ceramide. Elevated G_M3_, G_M2_, and G_M1_. |
| Gregorio *et al.* 2024 | *In vitro* | CBE-Oli-Neu cells (n=3) | HPTLC-MRM-MS | No quantitation reported. | DHC trending upwards but unchanged in CBE-Oli-Neu cells. |
| Mazzulli *et al.* 2011 | *In vitro* | *GBA1* knock-down cortical neurons (n=3) | LC-MS | No quantitation reported. | Ceramide unchanged. |
| Korkotian *et al.* 1999 | *In vitro* | CBE-treated rat hippocampal neurons (n=unknown) | TLC and photoluminescence | 2,073 counts per million at day 4 (0.95 fold change) and 7,248 counts per million at day 9 (0.88 fold change). 4 days: DHC, 2,525 cpm; G_M3_, 6,069 cpm; G_M2_, 1,757 cpm; G_M1_, 4,271 cpm; G_D1a_, 10,360 cpm; G_D1b_, 10,214 cpm; G_D3_, 12,902 cpm; G_T1b_, 13,227 cpm  9 days: DHC, 3,607 cpm; G_M3_, 6,350 cpm; G_M2_, 2,998 cpm; G_M1_, 5,702 cpm; G_D1a_, 10,498 cpm; G_D1b_, 9,938 cpm; G_D3_, 12,580 cpm; G_T1b_, 13,718 cpm | Unchanged [^3^H]ceramide following radioactive loading studies. No significant difference in [^3^H]GSLs in GD neurons vs controls following radio-labelling with [^3^H]Sphinganine. |
| Magalhaes *et al.* 2016 | *In vitro* | *GBA1* knock-down in SH-SY5Y cells (n=7) | UHPLC-MS | No quantitation reported. | Ceramide unchanged. 70% decrease in GCase activity also did not result in accumulated GlcCer. |
| Lunghi *et al.* 2022 | *In vitro* | CBE-iPSC-dopaminergic neurons (n=3) | HPTLC and radioactivity imaging | No quantitation reported. | Unchanged ceramide in iPSC-dopaminergic neurons following radioactive [^3^H]sphingosine loading. 2-fold increase of G_M3_, G_M1_, and G_D1a_, 50% increase in G_D1b_, and 25% increase of G_Q1b_ in iPSC-dopaminergic neurons. Overall, gangliosides were elevated following CBE treatment. |
| *Lunghi *et al.* 2022 | *In vitro* | CBE-cerebellar granule neurons (n=3) | HPTLC and radioactivity imaging | 50% reduced ceramide in cerebellar granule neurons. | Reduction of ceramide in cerebellar granule neurons. 50% increase in G_M3_ and G_M2_ and significant reduction of G_Q1b_ in cerebellar granule neurons. Overall, gangliosides were elevated following CBE treatment. |
| Campeau *et al.* 2009 | *In vitro* | CBE-mesenchymal stem cells (n=unknown) | HPLC-TMS | No quantitation reported. | Unchanged ceramide. |
| Du *et al.* 2015 | *In vitro* | SK-N-SH cells *GBA1* knock-down (n=3) | Immunocytochemistry | No quantitation reported. | Significantly reduced ceramide. |

CBE, conduritol B epoxide; CSF, cerebral spinal fluid; DHC, dihexosylceramide; ESI-MS/MS, electrospray ionisation tandem mass spectrometry; ESI-LC-MS/MS, electrospray ionisation liquid chromatography tandem mass spectroscopy; GCase, acid beta glucocerebrosidase; GD, Gaucher disease; GLC, gas liquid chromatography; GSL, glycosphingolipid; HPLC, high performance liquid chromatography; HPLC-MS/MS, high performance liquid chromatography tandem mass spectrometry; HPLC-TMS, high performance liquid chromatography tandem mass spectrometry; HPTLC, high performance thin layer chromatography; HPTLC-MRM-MS, high performance thin layer chromatography multiple reaction monitoring mass spectrometry; iPSC, induced pluripotent stem cell; KO, knock-out; LC-ESI-MS/MS, liquid chromatography electrospray ionisation tandem mass spectrometry; LC-MS, liquid chromatography mass spectrometry; LC-MS/MS, liquid chromatography tandem mass spectrometry; nLC-ESI-MS/MS, nano-scale liquid chromatography electrospray ionisation tandem mass spectrometry; Q-TOF MS/MS, quadrupole time-of-flight tandem mass spectrometry; Sap C, saposin C; THC, trihexosylceramide; TLC, thin layer chromatography; UHPLC-MS, ultra-high performance liquid chromatography mass spectrometry; UPLC-MS/MS, ultra performance liquid chromatography tandem mass spectrometry; WT, wildtype. *Indicates reports wherein more than one tissue/cell type was analysed within the same subheading and are therefore listed in duplicate.
